# 1,5-Dichloro-4,8-dinitro­anthraquinone

**DOI:** 10.1107/S1600536810029156

**Published:** 2010-07-31

**Authors:** Mahsa Armaghan, Mostafa M. Amini, Seik Weng Ng

**Affiliations:** a Department of Chemistry, General Campus, Shahid Beheshti University, Tehran 1983963113, Iran; b Department of Chemistry, University of Malaya, 50603 Kuala Lumpur, Malaysia

## Abstract

The ring skeleton of the title compound, C_14_H_4_Cl_2_N_2_O_6_, is close to planar (r.m.s. deviation of the carbon atoms 0.091 Å); the nitro goups are twisted with respect to the mean plane of the ring system by 70.8 (1) and 86.7 (2)°. The crystal studied  was found to be a merohedral twin, with a domain ratio of 0.61 (8):0.39 (8).

## Related literature

For dehydro­sulfurization by using anthraquinone-based catalysts, see: Nagai *et al.* (1993 ▶). For a related structure, see: Armaghan *et al.* (2010 ▶).
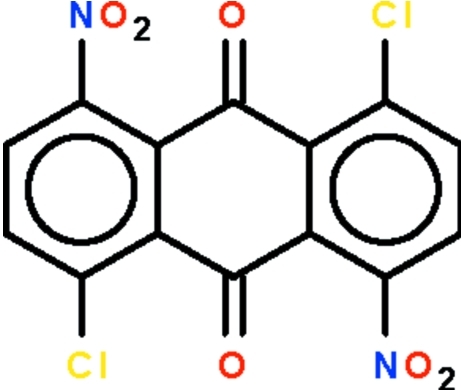


## Experimental

### 

#### Crystal data

C_14_H_4_Cl_2_N_2_O_6_
*M* *_r_* = 367.09
Monoclinic, 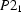

*a* = 5.9596 (6) Å
*b* = 11.3897 (11) Å
*c* = 9.8667 (9) Å
β = 93.519 (1)°
*V* = 668.47 (11) Å^3^
*Z* = 2
Mo *K*α radiation
μ = 0.53 mm^−1^
*T* = 100 K
0.12 × 0.12 × 0.12 mm

#### Data collection

Bruker SMART APEX diffractometer
Absorption correction: multi-scan (*SADABS*, Sheldrick, 1996 ▶) *T*_min_ = 0.940, *T*_max_ = 0.940
6510 measured reflections
3028 independent reflections
2779 reflections with *I* > 2σ(*I*)
*R*_int_ = 0.026

#### Refinement

*R*[*F* ^2^ > 2σ(*F* ^2^)] = 0.037
*wR*(*F* ^2^) = 0.095
*S* = 1.04
3028 reflections
218 parameters
1 restraint
H-atom parameters constrained
Δρ_max_ = 0.54 e Å^−3^
Δρ_min_ = −0.34 e Å^−3^
Absolute structure: Flack (Flack, 1983 ▶), 1402 Friedel pairs
Flack parameter: 0.39 (8)

### 

Data collection: *APEX2* (Bruker, 2009 ▶); cell refinement: *SAINT* (Bruker, 2009 ▶); data reduction: *SAINT*; program(s) used to solve structure: *SHELXS97* (Sheldrick, 2008 ▶); program(s) used to refine structure: *SHELXL97* (Sheldrick, 2008 ▶); molecular graphics: *X-SEED* (Barbour, 2001 ▶); software used to prepare material for publication: *publCIF* (Westrip, 2010 ▶).

## Supplementary Material

Crystal structure: contains datablocks global, I. DOI: 10.1107/S1600536810029156/kp2270sup1.cif

Structure factors: contains datablocks I. DOI: 10.1107/S1600536810029156/kp2270Isup2.hkl

Additional supplementary materials:  crystallographic information; 3D view; checkCIF report

## Figures and Tables

**Fig. 1.**
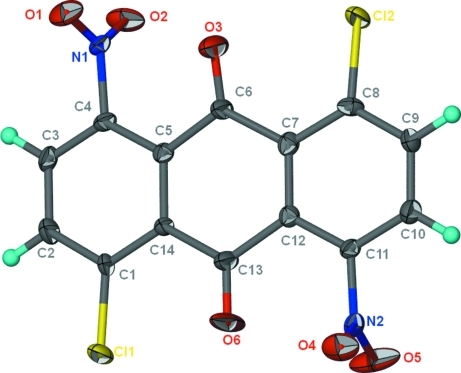
Displacement ellipsoid plot (Barbour, 2001) of I at the 70% probability level. Hydrogen atoms are drawn as spheres of arbitrary radius.

## supplementary crystallographic information

### Comment

The title compound (Scheme I, Fig. 1) belongs to a class of catalysts used for dehydrosulfurisation (Nagai *et al.*, 1993). We have embarked on a study of dehydrosulfurisation, and have recently reported the crysal structure of 1,8-dihydroxy-2,4,5,7-tetranitro-9,10-anthraquinone (Armaghan *et al.*, 2010). These compounds are synthesised by the reaction of fuming nitric acid on the substituted anthraquinone.

### Experimental

Fuming nitric acid (10 ml) was added dropwise to a solution of 1,5-dichloroanthraquinone (277 mg, 1 mmol) in concentrated sulfuric acid (5 ml). The mixture was kept at 333 K. After two hours, the mixture was poured into ice (100 g). The organic compound was collected and dried. Crystals suitable for X-ray analysis were obtained by recrystallisation from toluene; m.p. > 540 K.

### Refinement

Hydrogen atoms were placed in calculated positions (C–H 0.95 Å) and included in the refinement in the riding model approximation, with *U*(H) set to 1.2*U*_eq_(C).

### Crystal data

**Table:**

C_14_H_4_Cl_2_N_2_O_6_	*F*(000) = 368
*M**_r_* = 367.09	*D*_x_ = 1.824 Mg m^−^^3^
Monoclinic, *P*2_1_	Mo *K*α radiation, λ = 0.71073 Å
Hall symbol: P 2yb	Cell parameters from 2429 reflections
*a* = 5.9596 (6) Å	θ = 2.7–28.3°
*b* = 11.3897 (11) Å	µ = 0.53 mm^−^^1^
*c* = 9.8667 (9) Å	*T* = 100 K
β = 93.519 (1)°	Cube, yellow
*V* = 668.47 (11) Å^3^	0.12 × 0.12 × 0.12 mm
*Z* = 2	

### Data collection

**Table:**

Bruker SMART APEX diffractometer	3028 independent reflections
Radiation source: fine-focus sealed tube	2779 reflections with *I* > 2σ(*I*)
graphite	*R*_int_ = 0.026
ω scans	θ_max_ = 27.5°, θ_min_ = 2.1°
Absorption correction: multi-scan (*SADABS*, Sheldrick, 1996)	*h* = −7→7
*T*_min_ = 0.940, *T*_max_ = 0.940	*k* = −14→14
6510 measured reflections	*l* = −12→12

### Refinement

**Table:**

Refinement on *F*^2^	Secondary atom site location: difference Fourier map
Least-squares matrix: full	Hydrogen site location: inferred from neighbouring sites
*R*[*F*^2^ > 2σ(*F*^2^)] = 0.037	H-atom parameters constrained
*wR*(*F*^2^) = 0.095	*w* = 1/[σ^2^(*F*_o_^2^) + (0.0477*P*)^2^ + 0.339*P*] where *P* = (*F*_o_^2^ + 2*F*_c_^2^)/3
*S* = 1.04	(Δ/σ)_max_ = 0.001
3028 reflections	Δρ_max_ = 0.54 e Å^−^^3^
218 parameters	Δρ_min_ = −0.34 e Å^−^^3^
1 restraint	Absolute structure: Flack (Flack, 1983) parameter from 1402 Friedel pairs
Primary atom site location: structure-invariant direct methods	Flack parameter: 0.39 (8)

### Fractional atomic coordinates and isotropic or equivalent isotropic displacement parameters (Å^2^)

**Table:**

	*x*	*y*	*z*	*U*_iso_*/*U*_eq_	
Cl1	0.62144 (12)	0.00005 (6)	0.17540 (8)	0.02356 (19)	
Cl2	0.36066 (13)	0.69418 (6)	0.31405 (8)	0.0250 (2)	
O1	0.0933 (4)	0.3072 (2)	0.6211 (2)	0.0271 (5)	
O2	−0.1258 (4)	0.3417 (2)	0.4440 (2)	0.0277 (5)	
O3	0.2920 (4)	0.4726 (2)	0.4346 (2)	0.0339 (6)	
O4	1.1628 (4)	0.3759 (2)	0.0667 (2)	0.0280 (5)	
O5	0.9158 (4)	0.3677 (3)	−0.1002 (2)	0.0404 (7)	
O6	0.8065 (5)	0.2199 (2)	0.1277 (3)	0.0548 (9)	
N1	0.0447 (4)	0.3022 (2)	0.4997 (3)	0.0147 (5)	
N2	0.9756 (4)	0.3942 (2)	0.0136 (3)	0.0163 (5)	
C1	0.4613 (5)	0.0953 (3)	0.2635 (3)	0.0142 (6)	
C2	0.2957 (5)	0.0447 (3)	0.3375 (3)	0.0198 (6)	
H2	0.2742	−0.0379	0.3350	0.024*	
C3	0.1616 (5)	0.1141 (3)	0.4149 (3)	0.0162 (7)	
H3	0.0494	0.0799	0.4668	0.019*	
C4	0.1953 (5)	0.2341 (3)	0.4148 (3)	0.0136 (6)	
C5	0.3553 (5)	0.2890 (3)	0.3409 (3)	0.0126 (6)	
C6	0.3801 (5)	0.4179 (3)	0.3468 (3)	0.0181 (6)	
C7	0.5285 (4)	0.4765 (3)	0.2507 (3)	0.0150 (6)	
C8	0.5362 (5)	0.5992 (3)	0.2339 (3)	0.0153 (6)	
C9	0.6860 (5)	0.6510 (3)	0.1494 (3)	0.0170 (6)	
H9	0.6890	0.7340	0.1399	0.020*	
C10	0.8297 (5)	0.5827 (3)	0.0795 (3)	0.0183 (7)	
H10	0.9342	0.6178	0.0229	0.022*	
C11	0.8195 (5)	0.4621 (3)	0.0930 (3)	0.0132 (6)	
C12	0.6693 (5)	0.4075 (3)	0.1766 (3)	0.0132 (6)	
C13	0.6668 (5)	0.2752 (3)	0.1839 (3)	0.0224 (7)	
C14	0.4913 (4)	0.2177 (3)	0.2625 (3)	0.0121 (6)	

### Atomic displacement parameters (Å^2^)

**Table:**

	*U*^11^	*U*^22^	*U*^33^	*U*^12^	*U*^13^	*U*^23^
Cl1	0.0243 (3)	0.0116 (4)	0.0366 (4)	0.0019 (3)	0.0164 (3)	−0.0016 (3)
Cl2	0.0267 (4)	0.0132 (4)	0.0370 (4)	0.0049 (3)	0.0168 (3)	0.0022 (3)
O1	0.0307 (11)	0.0364 (13)	0.0147 (9)	0.0029 (10)	0.0041 (8)	−0.0053 (9)
O2	0.0209 (10)	0.0342 (13)	0.0276 (11)	0.0143 (10)	−0.0015 (9)	−0.0066 (10)
O3	0.0459 (13)	0.0198 (12)	0.0393 (13)	−0.0033 (10)	0.0285 (11)	−0.0054 (10)
O4	0.0191 (11)	0.0362 (14)	0.0284 (11)	0.0091 (9)	−0.0015 (9)	−0.0033 (10)
O5	0.0300 (12)	0.070 (2)	0.0210 (11)	0.0167 (12)	0.0000 (9)	−0.0185 (12)
O6	0.0656 (17)	0.0125 (12)	0.094 (2)	−0.0058 (12)	0.0693 (17)	−0.0087 (12)
N1	0.0140 (11)	0.0161 (13)	0.0147 (11)	−0.0012 (9)	0.0070 (9)	0.0004 (10)
N2	0.0167 (12)	0.0142 (13)	0.0188 (12)	−0.0046 (10)	0.0064 (10)	0.0013 (10)
C1	0.0172 (13)	0.0089 (14)	0.0168 (14)	−0.0012 (11)	0.0039 (11)	0.0026 (11)
C2	0.0225 (15)	0.0122 (15)	0.0252 (15)	−0.0012 (12)	0.0051 (12)	0.0029 (12)
C3	0.0133 (14)	0.0175 (18)	0.0183 (14)	−0.0027 (12)	0.0048 (11)	0.0054 (12)
C4	0.0109 (12)	0.0172 (16)	0.0133 (13)	0.0020 (11)	0.0036 (10)	−0.0002 (11)
C5	0.0140 (13)	0.0131 (15)	0.0106 (12)	−0.0012 (10)	0.0009 (10)	−0.0013 (10)
C6	0.0212 (14)	0.0118 (14)	0.0226 (14)	−0.0022 (11)	0.0104 (12)	−0.0021 (11)
C7	0.0144 (12)	0.0151 (17)	0.0154 (13)	−0.0035 (11)	0.0018 (10)	0.0006 (10)
C8	0.0108 (13)	0.0167 (16)	0.0186 (14)	0.0029 (11)	0.0025 (11)	−0.0036 (12)
C9	0.0208 (13)	0.0108 (14)	0.0198 (13)	−0.0027 (11)	0.0046 (11)	0.0010 (11)
C10	0.0232 (16)	0.0157 (17)	0.0166 (14)	−0.0026 (13)	0.0053 (12)	0.0008 (12)
C11	0.0136 (12)	0.0127 (15)	0.0137 (12)	−0.0025 (11)	0.0045 (10)	−0.0013 (10)
C12	0.0131 (12)	0.0129 (16)	0.0138 (13)	−0.0017 (12)	0.0013 (10)	0.0002 (10)
C13	0.0279 (15)	0.0142 (15)	0.0271 (16)	−0.0009 (12)	0.0170 (13)	−0.0024 (12)
C14	0.0109 (12)	0.0115 (16)	0.0142 (12)	0.0004 (10)	0.0039 (10)	0.0002 (10)

### Geometric parameters (Å, °)

**Table:**

Cl1—C1	1.716 (3)	C3—H3	0.9500
Cl2—C8	1.730 (3)	C4—C5	1.385 (4)
O1—N1	1.216 (3)	C5—C14	1.411 (4)
O2—N1	1.211 (3)	C5—C6	1.476 (5)
O3—C6	1.213 (4)	C6—C7	1.493 (4)
O4—N2	1.221 (3)	C7—C12	1.390 (4)
O5—N2	1.197 (3)	C7—C8	1.408 (5)
O6—C13	1.205 (4)	C8—C9	1.390 (4)
N1—C4	1.483 (4)	C9—C10	1.373 (5)
N2—C11	1.472 (4)	C9—H9	0.9500
C1—C2	1.388 (4)	C10—C11	1.382 (5)
C1—C14	1.405 (5)	C10—H10	0.9500
C2—C3	1.386 (5)	C11—C12	1.399 (4)
C2—H2	0.9500	C12—C13	1.509 (5)
C3—C4	1.382 (4)	C13—C14	1.492 (4)
			
O2—N1—O1	124.9 (3)	C12—C7—C8	118.3 (3)
O2—N1—C4	117.3 (2)	C12—C7—C6	118.8 (3)
O1—N1—C4	117.7 (2)	C8—C7—C6	122.9 (3)
O5—N2—O4	124.8 (3)	C9—C8—C7	121.2 (3)
O5—N2—C11	118.1 (3)	C9—C8—Cl2	115.9 (3)
O4—N2—C11	116.9 (2)	C7—C8—Cl2	122.8 (3)
C2—C1—C14	120.7 (3)	C10—C9—C8	120.3 (3)
C2—C1—Cl1	116.0 (3)	C10—C9—H9	119.9
C14—C1—Cl1	123.3 (2)	C8—C9—H9	119.9
C3—C2—C1	120.4 (3)	C9—C10—C11	118.8 (3)
C3—C2—H2	119.8	C9—C10—H10	120.6
C1—C2—H2	119.8	C11—C10—H10	120.6
C4—C3—C2	118.3 (3)	C10—C11—C12	122.1 (3)
C4—C3—H3	120.8	C10—C11—N2	116.0 (3)
C2—C3—H3	120.8	C12—C11—N2	121.9 (3)
C3—C4—C5	123.4 (3)	C7—C12—C11	119.2 (3)
C3—C4—N1	115.1 (3)	C7—C12—C13	122.1 (3)
C5—C4—N1	121.5 (3)	C11—C12—C13	118.7 (3)
C4—C5—C14	117.8 (3)	O6—C13—C14	122.4 (3)
C4—C5—C6	119.8 (3)	O6—C13—C12	119.4 (3)
C14—C5—C6	122.3 (3)	C14—C13—C12	118.2 (3)
O3—C6—C5	119.6 (3)	C1—C14—C5	119.3 (3)
O3—C6—C7	121.5 (3)	C1—C14—C13	122.2 (3)
C5—C6—C7	118.7 (3)	C5—C14—C13	118.5 (3)
			
C14—C1—C2—C3	−2.0 (4)	C9—C10—C11—N2	179.0 (2)
Cl1—C1—C2—C3	178.3 (2)	O5—N2—C11—C10	−87.6 (4)
C1—C2—C3—C4	0.9 (4)	O4—N2—C11—C10	87.7 (4)
C2—C3—C4—C5	0.3 (5)	O5—N2—C11—C12	92.1 (4)
C2—C3—C4—N1	179.8 (2)	O4—N2—C11—C12	−92.5 (3)
O2—N1—C4—C3	−94.0 (3)	C8—C7—C12—C11	2.7 (4)
O1—N1—C4—C3	81.0 (3)	C6—C7—C12—C11	−175.8 (3)
O2—N1—C4—C5	85.4 (3)	C8—C7—C12—C13	−177.7 (3)
O1—N1—C4—C5	−99.5 (3)	C6—C7—C12—C13	3.8 (4)
C3—C4—C5—C14	−0.4 (4)	C10—C11—C12—C7	−1.3 (4)
N1—C4—C5—C14	−179.8 (2)	N2—C11—C12—C7	179.0 (2)
C3—C4—C5—C6	−179.9 (3)	C10—C11—C12—C13	179.1 (3)
N1—C4—C5—C6	0.7 (4)	N2—C11—C12—C13	−0.6 (4)
C4—C5—C6—O3	13.8 (5)	C7—C12—C13—O6	−173.0 (3)
C14—C5—C6—O3	−165.6 (3)	C11—C12—C13—O6	6.6 (5)
C4—C5—C6—C7	−170.8 (2)	C7—C12—C13—C14	6.2 (5)
C14—C5—C6—C7	9.7 (4)	C11—C12—C13—C14	−174.2 (2)
O3—C6—C7—C12	163.6 (3)	C2—C1—C14—C5	1.9 (4)
C5—C6—C7—C12	−11.6 (4)	Cl1—C1—C14—C5	−178.4 (2)
O3—C6—C7—C8	−14.9 (5)	C2—C1—C14—C13	−179.7 (3)
C5—C6—C7—C8	169.9 (3)	Cl1—C1—C14—C13	0.0 (4)
C12—C7—C8—C9	−2.3 (4)	C4—C5—C14—C1	−0.7 (4)
C6—C7—C8—C9	176.2 (3)	C6—C5—C14—C1	178.8 (3)
C12—C7—C8—Cl2	176.9 (2)	C4—C5—C14—C13	−179.1 (3)
C6—C7—C8—Cl2	−4.6 (4)	C6—C5—C14—C13	0.4 (4)
C7—C8—C9—C10	0.3 (4)	O6—C13—C14—C1	−7.4 (5)
Cl2—C8—C9—C10	−178.9 (2)	C12—C13—C14—C1	173.4 (3)
C8—C9—C10—C11	1.2 (4)	O6—C13—C14—C5	171.0 (3)
C9—C10—C11—C12	−0.7 (5)	C12—C13—C14—C5	−8.2 (4)
